# Problematic Online Gaming in Autistic Adults With Mild Intellectual Disability in a Locked Rehabilitation Setting: A Service Evaluation of Triggers, Risk Escalation and a Structured Access Intervention

**DOI:** 10.1192/bjo.2026.11631

**Published:** 2026-06-30

**Authors:** Azmathulla Khan Hameed, Benny Daniel

**Affiliations:** Cygnet Hospital Harrow, London, United Kingdom

## Abstract

**Aims:**

- To characterise patterns of problematic online gaming in autistic adults with mild ID within a locked rehabilitation ward.
- To identify behavioural escalation triggers and staff interaction factors associated with gaming-related incidents.
- To evaluate the impact of a structured, formulation-driven “gaming access plan” on incidents and engagement outcomes.

**Methods:**

A prospective service evaluation was conducted in a locked rehabilitation inpatient service for adults with autism and mild ID. A structured pro-forma was implemented over a 12-week period to capture: (i) gaming access patterns (device type, duration, online vs offline), (ii) antecedents and triggers (including demand type and staff response style), and (iii) outcome measures (incidents, PRN administration, sleep disruption, and engagement in OT/rehabilitation activities).

Following baseline data collection, a standardised intervention was introduced comprising a personalised gaming plan (agreed daily access windows, visual time-limits, low-arousal staff scripts, and a supported transition routine), paired with consistent MDT implementation and alternative regulation activities. Descriptive statistics and pre/post comparisons were used.

**Results:**

Twelve Male patients were included (mean age 26 years). Online competitive multiplayer games were the most common activity (75%), and 67% of patients demonstrated daily gaming exceeding 4 hours. Gaming was predominantly used as an emotional regulation strategy and as an escape from demands.

Across baseline weeks, 61% of recorded incidents were temporally linked to gaming access or restriction. The most frequent escalation triggers were: (1) being asked to stop gaming mid-session (42%), (2) demand introduction during gaming (medication, hygiene, meetings) (28%), and (3) connectivity/game performance issues (e.g., lag, ranked loss) (18%). Escalation was more likely when limit-setting was delivered using direct instruction without warning compared with collaborative low-arousal approaches.

Following implementation of the structured gaming plan, total incidents reduced from 78 to 48 over comparable time periods (38% reduction). PRN use reduced by 29%, and OT engagement improved from 54% to 71% of planned sessions attended. Sleep disruption linked to late-night gaming reduced, with fewer recorded episodes of nocturnal dysregulation.

**Conclusion:**

Problematic online gaming is a clinically significant driver of distress and behavioural escalation within autistic adults with mild ID in locked rehabilitation. Incidents were most consistently associated with transitions, demand introduction, and loss of gaming-related control. A structured, autism-informed gaming plan with consistent low-arousal staff implementation was associated with meaningful reductions in incidents and PRN use, alongside improved rehabilitation engagement. This work supports the integration of gaming formulations into Positive Behaviour Support planning and highlights the need for further research into gaming disorder phenotypes in inpatient autism/ID services.

